# Enhanced production of recombinant proteins with *Corynebacterium glutamicum* by deletion of insertion sequences (IS elements)

**DOI:** 10.1186/s12934-015-0401-7

**Published:** 2015-12-29

**Authors:** Jae Woong Choi, Sung Sun Yim, Min Jeong Kim, Ki Jun Jeong

**Affiliations:** Department of Chemical and Biomolecular Engineering (BK Plus program), KAIST, 291 Daehakro, Yuseong-gu, Daejeon, 34141 Republic of Korea; Institute for the BioCentury, KAIST, 291 Daehakro, Yuseong-gu, Daejeon, 34141 Republic of Korea

**Keywords:** *Corynebacterium glutamicum*, IS element, FACS screening, Poly(3-hydroxybutyrate), γ-aminobutyrate

## Abstract

**Background:**

In most bacteria, various jumping genetic elements including insertion sequences elements (IS elements) cause a variety of genetic rearrangements resulting in harmful effects such as genome and recombinant plasmid instability. The genetic stability of a plasmid in a host is critical for high-level production of recombinant proteins, and in this regard, the development of an IS element-free strain could be a useful strategy for the enhanced production of recombinant proteins. *Corynebacterium glutamicum*, which is a workhorse in the industrial-scale production of various biomolecules including recombinant proteins, also has several IS elements, and it is necessary to identify the critical IS elements and to develop IS element deleted strain.

**Results:**

From the cultivation of *C. glutamicum* harboring a plasmid for green fluorescent protein (GFP) gene expression, non-fluorescent clones were isolated by FACS (fluorescent activated cell sorting). All the isolated clones had insertions of IS elements in the GFP coding region, and two major IS elements (IS*Cg1* and IS*Cg2* families) were identified. By co-cultivating cells harboring either the isolated IS element-inserted plasmid or intact plasmid, it was clearly confirmed that cells harboring the IS element-inserted plasmids became dominant during the cultivation due to their growth advantage over cells containing intact plasmids, which can cause a significant reduction in recombinant protein production during cultivation. To minimize the harmful effects of IS elements on the expression of heterologous genes in *C. glutamicum*, two IS element free *C. glutamicum* strains were developed in which each major IS element was deleted, and enhanced productivity in the engineered *C. glutamicum* strain was successfully demonstrated with three models: GFP, poly(3-hydroxybutyrate) [P(3HB)] and γ-aminobutyrate (GABA).

**Conclusions:**

Our findings clearly indicate that the hopping of IS elements could be detrimental to the production of recombinant proteins in *C. glutamicum*, emphasizing the importance of developing IS element free host strains.

**Electronic supplementary material:**

The online version of this article (doi:10.1186/s12934-015-0401-7) contains supplementary material, which is available to authorized users.

## Background

*Corynebacterium glutamicum* is a Gram-positive bacterium which is non-pathogenic, non-sporulating, and generally-regarded as safe (GRAS) [1, 2]. Due to its safety and process performance, it has been used as one of the best amino acid producers in industry and for the production of various bio-based chemicals such as poly(3-hydroxybutyrate) [P(3HB)], γ-aminobutyrate, cadaverine, and putrescine [3, 4]. Recently, *C. glutamicum* has also been regarded as a potential emerging host for the production of many recombinant proteins because it can secrete proteins in a non-pathogenic background, which can be advantageous in biopharmaceutical protein production with subsequent downstream protein purification processes [5, 6]. However, although *C. glutamicum* is an efficient host strain, several properties remain to be improved. In particular, mobile genetic elements (MGEs) are problematic for bacterial genome and plasmid stability [7, 8]

After the discovery of the transposon in 1950 [9], mobile genetic elements (MGEs) have been studied in many organisms including bacteria. MGEs are the key factors in genome rearrangement and evolution, and they have important roles in the adaptations of bacteria to their environment by inversion, recombination, and insertion [10, 11]. There are various classes of bacterial MGEs: insertion sequence elements (IS elements), prophages, bacterial interspersed mosaic elements, miniature inverted-repeat transposable elements, repetitive extragenic palindromic elements sequences, etc. [12]. In *C. glutamicum*, a few studies on the effects of MGEs on the bacterial cells and their protein productivities have been reported. The *C. glutamicum* prophage CGP3 has an increased copy number under stress conditions including UV light, genotoxic agent, or DNA damage, and bacterial cells containing multiple prophage DNA molecules were more prone to cell lysis [7]. In addition, the enhanced production of recombinant proteins and high transformation efficiency were achieved in prophage-free *C. glutamicum* [8]. Also, there was an attempt to construct chassis strain of *C. glutamicum* by removing 36 unnecessary gene clusters which include some of IS elements [13].

As another important factor, IS elements have been studied in many bacterial species [14, 15]. IS elements usually encode transposases and terminal inverted repeats (TIR) which determine the IS family. IS elements move by encoding transposases and insert into specific sequences by the TIR [16]. Most IS elements have no activity by themselves, but when they are inserted into a gene, they change the gene expression by activation or disruption [17]. They induce large duplication and inversion, and large genomic rearrangement making the genome unstable [18–20]. Additionally, IS elements can cause a significant instability of plasmid that carry heterologous genes, which cause the reduced production of the encoded proteins. For example, in the production of a DNA vaccine which is produced from a plasmid in *E. coli*, the insertion of IS elements from the chromosome into the plasmid reduced the productivity [21, 22]. In another case, cells harboring IS inserted plasmids had a better growth rate than that of cells harboring non IS inserted plasmids, which encoded the target recombinant protein, and this difference in growth rate induced about a 25 % reduction in productivity [23]. Thus, a stable and efficient host strain that is free from the adverse effects of IS elements is highly desirable for both laboratory and industrial applications.

In this study, we first sought to identify the major IS elements in *C. glutamicum*, of which insertion can cause the disruption of genes in plasmid. Using a FACS-based high throughput screening system with the green fluorescent protein (GFP) as the reporter protein, the major IS element could be identified. Next, we tried to engineer *C. glutamicum* in which the major IS element was deleted in the chromosomal DNA and, using the engineered *C. glutamicum*, we demonstrated the enhanced production of recombinant proteins, poly(3-hydroxybutyrate) [P(3HB)], and γ-aminobutyrate (GABA).

## Results

### Isolation of IS elements by FACS and their identification

**Fig. 1:**
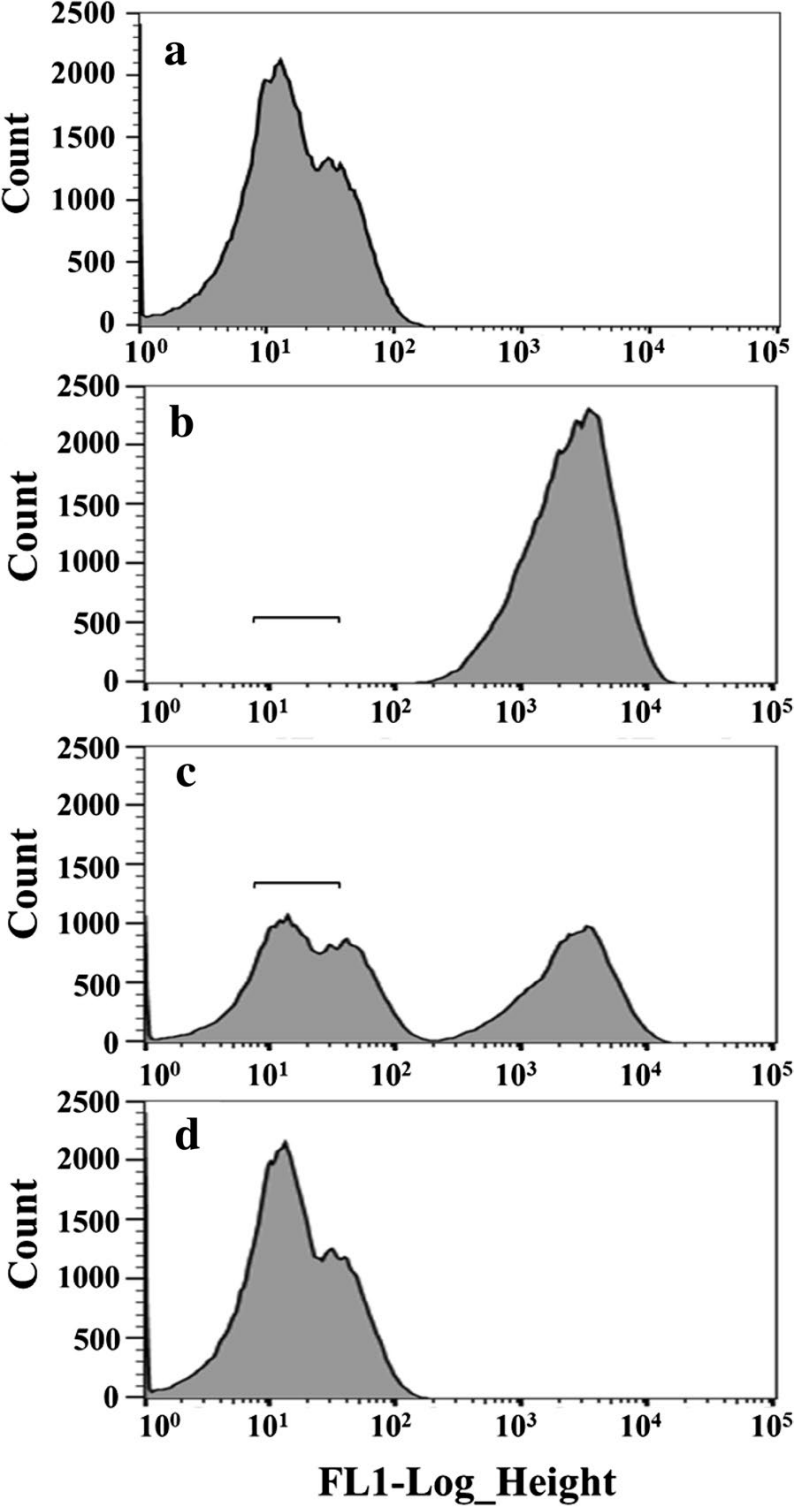
FACS sorting of low fluorescent cells in the cultivation of wild type *C. glutamicum* harboring pCES-H36-GFP. **a** The histogram of wild type *C. glutamicum* harboring pCES-NMCS (negative control). **b**, **c**, **d** The histogram of wild type *C. glutamicum* harboring pCES-H36-GFP (**b**), the 1st round sorted cells (**c**) and the 2nd round sorted cells (**d**). The *bars* indicate the sorting region

To isolate the major IS elements in *C. glutamicum*, a GFP reporter system (pCES-H36-GFP) was constructed in which GFP expression was under a strong constitutive promoter (P_H36_). If the IS element is inserted in the GFP coding sequence, cells cannot produce functional GFPs, and those cells can be selectively sorted by a high–speed FACS sorter. After flask cultivation, the fluorescence intensity of the *C. glutamicum* harboring either pCES-NMCS or pCES-H36-GFP was analyzed by flow cytometry. Cell harboring pCES-NMCS which was used as negative control had no fluorescence intensity (Fig. 1a). Most cells harboring pCES-H36-GFP showed high fluorescence intensities (mean fluorescent intensity ≅8000); however, there was also a small population (about ~0.6 %) with no fluorescence (Fig. 1b). To identify the cells in this region, cells with no fluorescence were selectively sorted by the FACS sorter. In the first round of sorting, approximately 3.10 × 10^7^ cells were screened, and the bottom 0.61 % of cells (~1.9 × 10^5^ cells) were sorted. The sorted cells were cultivated and used for the next round of sorting. After the first round of sorting, the cell population showing no fluorescence was enriched (about 50 % of the total population) (Fig. 1c), and these non-fluorescent cells (~2.1 × 10^5^ cells) were also selectively sorted. After the second round of sorting, most of the population was found to have no fluorescent signal (Fig. 1d), and we concluded that the non-fluorescent cells were successfully enriched.

**Fig. 2:**
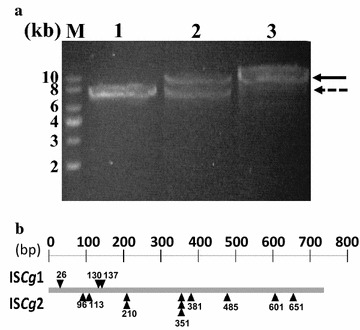
Analysis of the isolated cells by FACS screening. **a** Confirmation of plasmids by agarose gel electrophoresis. *Lane M* represents DNA size markers (kb). *Lanes 1* represents plasmid from the original cell cultivation harboring pCES-H36-GFP. *Lanes 2* and *3* represent plasmids from the 1st round sorted cells and the 2nd round sorted cells. The *dashed* and *solid arrows* indicate the correct pCES-H36-GFP and IS element-inserted plasmid, respectively. **b** Location of IS element insertion in e*gfp*. *Gray bar* indicates the open read frame of e*gfp*. *Arrows* indicate the IS element insertion site. *Upper* and *lower region* represent IS*Cg*1 and IS*Cg*2, respectively. *Double* or *triple triangles* means double or triple clones

In each round of FACS screening, plasmids were prepared from the sorted cells, and after digestion with the *Bam*HI restriction enzyme, they were analyzed on agarose gels. In the original population, a single band for the plasmid was clearly observed for which the size coincided well with that of pCES-H36-GFP (~6.6 kb) (lane 1 of Fig. 2a). However, in the samples after the first round of sorting, another distinct band was also observed at approximately 8 kb. The ratio of the densities between the two bands was about 50:50 (lane 2 of Fig. 2a). After the second round of sorting, it was clearly observed that the 8 kb-long plasmid became dominant in the sorted cells (lane 3 of Fig. 2a). After the second round of sorting, 14 clones were randomly selected, and GFP expression in each clone was analyzed by SDS-PAGE. As expected, all 14 clones did not produce GFP (Additional file 1: Figure S1). We performed sequencing experiment for the DNA from 72-bp upstream of H_36_ promoter to 71-bp downstream of GFP stop codon. The sequences of the GFP coding regions for the 14 clones were determined by sequencing which showed that all 14 clones had insertions of IS elements at various positions in the GFP coding region (Fig. 2b). However, we could not find any insertion of IS element in promoter region. From the sequencing analysis, it was found that two types of IS elements were inserted, and the IS elements were identified to be part of the IS*Cg1* and IS*Cg2* family, by searching the *C. glutamicum* genome database. Among the 14 clones, 11 clones contained insertions from the IS*Cg2* family, and the rest (3 clones) contained insertions from the IS*Cg1* family (Fig. 2b).

### Effect of gene deletion by IS element insertion on cell growth

**Fig. 3:**
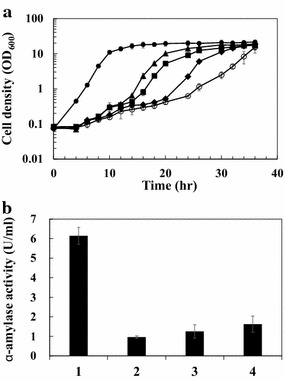
Determination of cell population change during co-culture of *C. glutamicum* wild type harboring pCES-H36-porBss-Amy and pCES-H36-porBss-IS-Amy. **a** Growth curves during the cultivation. *Open circles* and *closed circles* represent *C. glutamicum* harboring pCES-H36-porBss-Amy and *C. glutamicum* harboring pCES-H36-porBss-IS-Amy, respectively. *Triangles*, *squares* and *diamonds* represent co-culture of *C. glutamicum* (pCES-H36-porBss-IS-Amy) and *C. glutamicum* (pCES-H36-porBss-Amy) with the ratio of 1:10^3^, 1:10^4^, and 1:10^5^, respectively. **b** α-Amylase activity of the co-culture. 1, 2, 3, and 4 represent enzyme activity in the cultivation of *C. glutamicum* (pCES-H36-porBss-Amy), co-culture with the ratio of 1:10^3^, 1:10^4^, and 1:10^5^, respectively

Cells containing IS element-inserted plasmid cannot produce the target protein, and in many reports, it is well recognized that cells producing recombinant proteins have slower cell growth (very poor growth in some cases) compared to that of non-producing cells due to the metabolic burden or other problems including the toxicity of the proteins to the host cells, etc. [23]. Particularly, in large-scale production which generally needs long operation time, the fast growth of non-producing cells can be a very serious issue due to a significant decrease in protein productivity by the overgrowth of non-producing cells. To confirm this deleterious effect of IS element insertion on cell growth, two expression systems were constructed: (1) α-Amylase gene expression under the constitutive P_H36_ promoter (pCES-H36-porBss-Amy) and (2) IS element-inserted version (pCES-H36-porBss-IS-Amy) in which IS*Cg1* was inserted in the middle of signal sequence. *C. glutamicum* harboring each plasmid were cultivated in flasks, and the growth of each strain was compared. Additionally, IS element-inserted *C. glutamicum* cells were mixed with *C. glutamicum* producing α-amylase cells at different ratios (1:10^3^, 1:10^4^, and 1:10^5^), and their growth rates were also compared. As shown in Fig. 3, *C. glutamicum* harboring the pCES-H36-porBss-Amy grew slowly (specific growth rate, *μ* = 0.268 ± 0.052 h^−1^) after a long lag phase and reached stationary phase around 36 h. In contrast, the IS element-inserted version (*C. glutamicum* harboring pCES-H36-porBss-IS-Amy) grew much faster (*μ* = 0.552 ± 0.012 h^−1^) after a short lag phase and reached stationary phase much earlier (around 12 h). In the co-culture with the IS element-inserted cells and α-amylase-producing cells, the overall growth rates were also increased as the ratio of the IS element-inserted cells increased from 1:10^5^ to 1:10^3^ (*μ* = 0.305 ± 0.021, *μ* = 0.405 ± 0.041, and *μ* = 0.498 ± 0.039, respectively). In the co-culture experiments, the change in population during cultivation was analyzed by PCR with individual clones. At the end of each cultivation (at 36 h), cells were spread onto an agar plate, and 30 colonies from each plate were randomly selected, and the plasmids were confirmed by PCR experiment. For both ratios of 1:10^3^ and 1:10^4^, all the colonies were IS element-inserted clones, and for the ratio of 10^5^, IS element-inserted clones were also the major population (86 %) (data not shown). We also checked α-amylase activity in all cultivations at 36 h time-point, and we found that the cultivation of *C. glutamicum* harboring pCES-H36-porBss-Amy (without mixing with IS element-inserted clones) showed much higher activity (6.14 ± 0.44 U/mL) than those of other mixed cultures (Fig. 3b). In the cultivation of cells containing pCES-H36-porBss-Amy only, the possible insertion of IS element into plasmid was also analyzed by PCR but we could not find any insertion in the plasmid during the cultivation (data not shown). These results clearly show that the insertion of the IS element causes a significant decrease in protein productivity by the overgrowth of non-producing cells, and it is postulated that the removal of the IS element in the chromosome could be an important strategy for enhancing the production of target molecules in *C. glutamicum*.

### Deletion of the IS families (IS*Cg1* and IS*Cg2*)

It has been reported that four copies of IS*Cg1* (IS*Cg1a*, IS*Cg1b*, IS*Cg1c,* and IS*Cg1d*) and five copies of IS*Cg2* (IS*Cg2b*, IS*Cg2c*, IS*Cg2d*, IS*Cg2e*, and IS*Cg2f*) exist in the chromosomal DNA of *C. glutamicum* [24]. Each of the IS elements was deleted sequentially, and finally, two strains, WJ004 with the deletion of IS*Cg1* family and WJ008 with the deletion of IS*Cg2* family, were developed. The successful deletion of each IS element was clearly confirmed by PCR (Additional file 2: Figure S2). Interestingly, during the deletion of the IS elements, we discovered new information about the copy number and location of the IS elements. First, only four copies of IS*Cg2* were found in *C. glutamicum* ATCC 13032. IS*Cg2e* which was reported to exist at position (2317958..2319472; GenBank No. NC_006958.1) was not found in this study. Actually, after deletion of the four IS elements (IS*Cg2b*, IS*Cg2c*, IS*Cg2d*, and IS*Cg2f*) in *C. glutamicum*, no PCR products for the IS*Cg2* family members were detected which means IS*Cg*2 was not present in the chromosome any more. Second, we also found that IS*Cg1c,* one of the IS*Cg1* family members, does not exist in the reported position (611123..612433; GenBank No. NC_006958.1); however, we found a new IS*Cg1* element at another position (613777..615087; GenBank No. NC_006958.1). This IS element was named as IS*Cg1e* which was also deleted in the WJ004 strain. All IS elements deleted in this study are listed in Table 1. After developing both IS element-deleted mutants, the possible jumping of IS elements to other positions, was also checked by PCR, but we could not find any more IS*Cg*1 (in WJ004) and IS*Cg*2 (in WJ008) (data not shown).

**Table 1:** List of deleted IS elements in *C. glutamicum* ATCC13032 in this study

IS family	Copy number	IS element	Position
IS*Cg1*	4	IS*Cg1a*	1115955..1117265
		IS*Cg1b*	2595722..2597032
		IS*Cg1d*	2481196..2482506
		IS*Cg1e* ^a^	613777..615087
IS*Cg2* ^b^	4	IS*Cg2b*	3005658..3007172
		IS*Cg2c*	2716287..2717801
		IS*Cg2d*	2317958..2319472
		IS*Cg2f*	192954..194468

^a^New IS element found in this study. This gene was not reported in both *C. glutamicum* genome sequences deposited with GenBank Accession numbers NC_003450 and NC_006958.1

^b^IS*Cg2e* in IS*Cg2* family was absent in *C. glutamicum* ATCC13032 in our lab stock

### Enhanced production of recombinant protein in the IS element-deleted strain

**Fig. 4:**
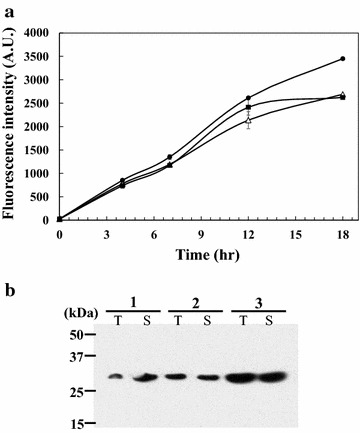
Comparison of GFP production in IS element-deleted mutants. **a** Analysis of fluorescence intensity in the cell by FACS. *C. glutamicum* WJ004 harboring pCES-H36-GFP and *C. glutamicum* WJ008 harboring pCES-H36-GFP are represented by *squares* and *circles*, respectively. *Open triangles* represent wild type *C. glutamicum* harboring pCES-H36-GFP. **b** Analysis of GFP production by Western blotting. *Lanes 1*, *2*, and *3* represent the protein sample from wild type *C. glutamicum*, WJ004, and WJ008 harboring pCES-H36-GFP at 18 h. *Lanes T* and *S* represent total protein fraction and soluble protein fraction. Protein sample was prepared from the same cell concentration which was normalized to OD_600_ of 4

To evaluate the effect of the IS element deletions on the production of recombinant proteins, the expression of GFP in the engineered strains (WJ004 and WJ008) was examined. In flask cultivations, cell growth and fluorescence intensity of both strains harboring pCES-H36-GFP were measured and we found all strains showed similar growth rates as that of wild-type cell harboring same plasmid (Additional file 3: Figure S3). Among the three strains, the WJ004 strain, in which the IS*Cg1* element was deleted, had almost the same production yield as the wild type *C. glutamicum*; however, the WJ008 strain, in which the IS*Cg2* elements were deleted, had an improved production yield as high as 130 % compared with WJ004 and wild type *C. glutamicum* (Fig. 4a). The higher production of GFP in WJ008 strain than other strains was also confirmed by Western blotting (Fig. 4b). In addition, the portion of non-fluorescent cells in each IS element-deleted strain harboring pCES-H36-GFP were also analyzed by FACS. In this analysis, WJ004 showed similar portion of non-fluorescent cells compared with that of wild type cell, but the portion of non-fluorescent cells in the cultivation of WJ008 decreased relatively by 66 %. These results indicate that the IS*Cg2* element is a more critical factor causing adverse effects on the productivity of heterologous proteins by insertion into the target gene, and this result is also agreed with the FACS screening result in which the IS*Cg2*–inserted clones were the major population (11 out of 14 clones) in the isolated cells.

### Enhanced production of P(3HB) and GABA in the IS element-deleted strain

**Fig. 5:**
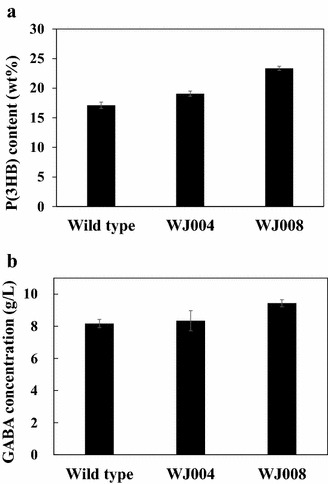
Production of a secondary metabolite. **a** Production of P(3HB) in the wild type *C. glutamicum*, WJ004 and WJ008 harboring pCES-H36-PhaCAB. All samples were prepared after 24 h of cultivation. **b** Production of GABA in the wild type *C. glutamicum*, WJ004 and WJ008 harboring pHGmut. All samples were prepared after 72 h of cultivation

The production of useful metabolites in *C. glutamicum* can be achieved by the expression of biosynthesis genes. However, if the IS element is inserted into the biosynthesis genes, those genes cannot be expressed, and the productivity of the target metabolites subsequently decreases. In this regard, the use of an IS element-deleted mutant can be beneficial for the biosynthesis of metabolites. To demonstrate this beneficial concept, the production of (1) poly(3-hydroxybutyrate) [P(3HB)], biodegradable polymers [25], and (2) gamma-aminobutyric acid (GABA), the bioactive component in various foods and pharmaceutical products [26], were examined with the IS element-deleted mutants. For the biosynthesis of P(3HB) in *C. glutamicum*, *C. glutamicum* wild type, WJ004 and WJ008 strains were transformed with the plasmid, pCES-H36-PhaCAB, in which the expressions of three genes (*phaC, phaA and phaB*) from *Ralstonia eutropha* were under the constitutive P_H36_ promoter. In these cultivations, all cells showed almost same growth profiles (Additional file 4: Figure S4). After flask cultivation for 24 h, the P(3HB) content in each strain was analyzed by gas chromatography. As shown in Fig. 5a, the use of the WJ008 strain resulted in a higher content (23.4 ± 0.36 wt%) than that in the WJ004 and wild type *C. glutamicum* strain (19.1 ± 0.44 wt% and 17.1 ± 0.55 wt%, respectively). For the biosynthesis of GABA in *C. glutamicum*, the plasmid pHGmut, in which the expression of glutamate decarboxylase (*gadB*) from *Escherichia coli* is under the constitutive P_H36_ promoter, was transformed into the WJ004 and WJ008 strains, and were cultured for 72 h. A wild type *C. glutamicum* grew a little faster than others, but all strains reached almost same optical density after 36 h cultivation (Additional file 4: Figure S4). The GABA content in each strain was analyzed by liquid chromatography. Like in the previous results, higher production of GABA (9.43 ± 0.52 g/L) could be obtained in the WJ008 strain than in the WJ004 and wild type *C. glutamicum* strain (8.34 ± 0.62 and 8.17 ± 0.66 g/L, respectively) (Fig. 5b).

### Effect of IS element deletion on transformation efficiency

**Fig. 6:**
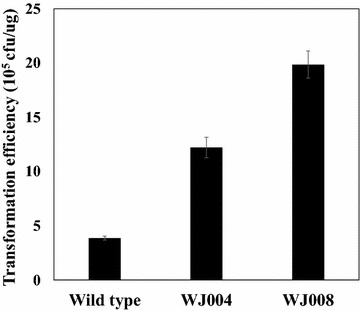
Transformation efficiency of the wild type *C. glutamicum*, WJ004 and WJ008. All *error bars* represent standard deviations of six-times repeated experiments

An earlier study [8] showed that the deletion of prophages in the chromosome of *C. glutamicum* (ATCC 13032) resulted in an increase in the transformation efficiency due to the deletion of the restriction modification (RM) system present in the prophage gene. Takahashi N et al. [27] also reported that the movement of the restriction modification system in *Escherichia coli* was linked with an IS element. In this regard, the deletion of IS elements may also have a positive effect on the transformation efficiency; thus, the effect of the IS element deletion was examined on the transformation efficiency as another characteristic of the IS element-deleted strains. The two IS element deleted mutants (WJ004 and WJ008) and wild-type *C. glutamicum* strain were transformed with the same plasmid (pCES-H36-GFP), and their transformation efficiencies were compared by checking the colony forming units (CFUs) on selective agar plates. As shown in Fig. 6, the WJ004 and WJ008 strains had about a four- and six-fold higher transformation efficiency (12.2 ± 0.94 × 10^5^ cfu/μg and 19.8 ± 1.2 × 10^5^ cfu/μg) than that of the wild type strain (3.86 ± 0.18 × 10^5^ cfu/μg), respectively.

## Discussion

Maximizing the production yield of the target product is crucial in the industrial biotechnology field, and for microbial production of recombinant proteins, it is necessary to construct a stable gene expression system. On a few occasions, the disruption of the heterologous gene occurs when IS elements are inserted inside the gene coding region [28, 29]. The disrupted gene cannot be translated into the desired protein, so it can decrease the production yield of the recombinant protein. In addition, the overgrowth of non-producing cells can cause a serious problem in the overall productivity in industrial cultivation. In this study, we isolated the major IS elements with a FACS-based high throughput screening system, and we also developed *C. glutamicum* strains in which the major IS elements were completely removed. So far, there have been only a few reports on the IS elements in *C. glutamicum*, and all of them were focused on the isolation of IS elements and identification of their locations in the genome. However, the adverse effects of IS elements on recombinant protein production in *C. glutamicum* has not been investigated yet. As shown in Fig. 3, *C. glutamicum* harboring the IS-inserted plasmid, in which the target protein cannot be produced, had a much higher cell growth compared with cells harboring intact plasmids in which the target proteins were overproduced. This difference in cell growth can be very serious in industrial fermentations which generally need long cultivation times. Even though the possibility of an IS element insertion event is very low at about 1 × 10^−5^ [28, 30, 31], IS element-inserted cells that can grow much faster become dominant during the fermentation, and consequently, the production yield can be significantly reduced. If the target molecules are not favorable to the *C. glutamicum* cells, this harmful effect on cell growth and productivity can be a much more serious issue.

The deletion of IS elements in a host strain can be one strategy to minimize the harmful effect of IS elements on the production of recombinant proteins. However, in most bacteria including *C. glutamicum*, IS elements are present in multiple copies [24, 32], and there have been a few attempts to develop a minimized genome strain in which all the IS elements were removed [33, 34]. However, although there has been significant progress in genome editing technology in the last decade, genome minimizing is still laborious and challenging work. Additionally, not all genome-minimized strains have always shown better performance in cell growth and protein productivity compared to the wild-type strains [23, 29]. One research group attempted to prevent IS element insertion using the ‘Clean Genome’ of the *E. coli* MDS42 strain in which 15 % of the genome including the IS elements is reduced [29]. However, the MDS42 strain had a lower expression level of GFP because the reduced genome made the cells very unstable, and it also took more time to maximize the growth rate given the extra metabolic load [29]. Thus, instead of deleting of all the IS elements, the deletion of specific IS elements is necessary to engineer a *C. glutamicum* strain with a high growth rate and productivity, and for this purpose, it is necessary to isolate the major IS elements which are most frequently inserted into heterologous genes. For this, FACS screening strategy was introduced, and we could successfully isolate two important IS elements (IS*Cg1* and IS*Cg2*). Of the two IS elements, the IS*Cg2* was more dominant than that of IS*Cg1* (Fig. 2b), and this means IS*Cg2* has a relatively higher transposable activity than that of IS*Cg1*, and the deletion of IS*Cg2* can be more effective in the production of target molecules than deletion of IS*Cg1* as we clearly showed with the three target biomolecules, GFP, P(3HB) and GABA. In the FACS screening, only 0.6 % cells in the total population which showed no fluorescent signals, was sorted (Fig. 1), but the WJ008 strain harboring same plasmid (pCES-H36-GFP) exhibited a very high increase (about 30 %) in the GFP production compared with that of wild type strain (Fig. 4). We consider that the sorting region for FACS screening (0.6 % in the total population) in the Fig. 1b did not cover all non-fluorescent population shown in Fig. 1a. This means that the actual portion of non-producing cells were much higher than 0.6 % in the total population, and the deletion of IS element could eliminate much more non-fluorescent cells than the cells in the sorted region (0.6 %). In addition, it can be considered that the removal of non-fluorescent cells which could grow much faster, might result in the better growth of the fluorescent cells, and much higher production yield than the removed amount of non-fluorescent cells could be obtained. Likewise, the positive effect of IS element deletion on target gene expression has also been reported in *E. coli* [23]. Even though IS hopping on target genes occurred very rarely (10^−8^/gene/h), IS-free *E. coli* showed 120 and 125 % enhanced productivities [23].

Additionally, we would like to emphasize the usefulness of the FACS-based high throughput screening method for the isolation of IS elements. In other methods that using promoterless antibiotics resistance genes [35] and other lethal genes [36, 37] as selection markers, cells can be selected when a copy of an IS element is inserted into the selection markers, and the IS element can be identified from the isolated clone. However, those methods have a limitation in the screening size and is not suitable for the screening of large sized libraries in general. When we consider the low possibility of an IS element insertion event, the FACS-based screening method, which can screen a big population (>10^8^ cells) in a short time, could be a more powerful tool as shown here.

As an additional advantage, we successfully confirmed that the deletion of the IS element had a highly positive effect on the transformation efficiency (Fig. 6). Transformation efficiency has a correlation with a restriction modification system (RM system), and there are reports that IS elements guide RM systems [27, 38, 39]. It is also known that IS element-RM system complexes can integrate into a plasmid or chromosomal DNA, which causes instability in the plasmid [21–23]. By deleting the IS elements, the activity of the RM system can be diminished, and as a consequence, the transformation efficiency can increase through the higher stability of the plasmid.

## Conclusions

In this study, we isolated the major IS elements in *C. glutamicum* and successfully developed two IS element-deleted *C. glutamicum* strains to minimize the harmful effect of IS elements on the production of target biomolecules. Using FACS-based high throughput screening, the major IS element, which can insert into heterologous genes in a plasmid most frequently, was successfully isolated. Using three different molecules, we also successfully showed that the production yield of the target biomolecules were significantly improved by deletion of the major IS element. To the best of our knowledge, this is the first report on the isolation of major IS elements using FACS screening strategy. Although we did not try in this study, it may be worth to combine the deletions of both IS elements (IS*Cg*1 and IS*Cg*2) in one strain. The development of that mutant and its use for the overproduction of recombinant proteins in the large-scale cultivation will be the future work of ours. Our study presents the IS element could be harmful to the production of recombinant proteins in *C. glutamicum*, emphasizing the importance of the development of IS-deleted host strains. Thus, we believe that the IS element deleted strain with its enhanced genetic stability can be a robust and appropriate host for the production of recombinant proteins and many other biomolecules.

## Methods

### Bacterial strains and growth conditions

The bacterial strains used in this study are listed in Table 2. *E. coli* XL1-blue was used as the host for gene cloning and plasmid maintenance. *C. glutamicum* ATCC 13032 was used for isolation of the major IS elements and the production of recombinant proteins. *E. coli* was cultivated in Luria–Bertani (LB) medium (BD, Franklin Lakes, New Jersey) at 37 ^o^C. *C. glutamicum* was cultivated at 30 ^o^C in brain heart infusion (BHI) medium (BD) with or without 2 % (*w*/*v*) d-glucose and shaking at 200 rpm. Additionally, a defined medium containing 20 g/L d-glucose, 3 g of K_2_HPO_4_, 1 g of KH_2_PO_4_, 2 g of urea, 10 g of (NH_4_)_2_SO_4_, 2 g of MgSO_4_, 200 μg of biotin, 5 mg of thiamine, 10 mg of calcium pantothenate, 10 mg of FeSO_4_, 1 mg of MnSO_4_, 1 mg of ZnSO_4_, 200 μg of CuSO_4_, and 10 mg of CaCl_2_ per liter [5] and modified GP medium [26] were used. For the co-culture experiment, wild-type *C. glutamicum* harboring pCES-H36-porBss-Amy or pCES-H36-porBss-IS-Amy were inoculated into separate BHI medium. After 24 h, the cells were harvested at an OD_600_ of 4.0 and resuspended in 1 mL of phosphate-buffered saline (PBS). The resuspended cells were transferred into fresh defined medium at pCES-H36-porBss-IS-Amy:pCES-H36-porBss-Amy ratios of 1:10^3^, 1:10^4^, and 1:10^5^. Then, cells were cultivated for 27 h at 30 ^o^C. For the heterologous protein production assay, cells were inoculated into BHI medium. After overnight cultivation, fully grown cells were transferred into a 250 mL flask containing 50 mL of fresh BHI medium and cultivated for 24 h. To produce poly(3-hydroxy butyrate) [P(3HB)] and γ -aminobutyric acid (GABA), *C. glutamicum* was first inoculated into BHI medium, and after cultivation for 24 h, cells were transferred into a 250 mL flask containing 100 mL of fresh BHI medium containing 2 %(*w*/*v*) d-glucose or modified GP medium for the production of P(3HB) or GABA, respectively. All experiments were performed in triplicate. In all cultivations, kanamycin (Km, 25 μg/mL) was added to the culture medium as the sole antibiotic.

**Table 2:** Bacterial strains and plasmids used in this study

	Relevant characteristics	Reference or source
Strain		
*E. coli*		
XL1-blue	*recA1 endA1 gyrA96 thi*-*1 hsdR17 supE44 relA1 lac [F′ proABlacIq ZΔM15 Tn10 (Tet* ^*r*^ *)]*	Stratagene^a^
*C. glutamicum*		
ATCC 13032	Biotin-auxotrophic wild type	ATCC
WJ001	ATCC 13032 with in frame deletion of IS*Cg2* family (IS*Cg1a*)	This study
WJ002	ATCC 13032 with in frame deletion of IS*Cg2* family (IS*Cg1a*, IS*Cg1b*)	This study
WJ003	ATCC 13032 with in frame deletion of IS*Cg2* family (IS*Cg1a*, IS*Cg1b*, IS*Cg1c*)	This study
WJ004	ATCC 13032 with in frame deletion of IS*Cg2* family (IS*Cg1a*, IS*Cg1b*, IS*Cg1c,* IS*Cg1e*)	This study
WJ005	ATCC 13032 with in frame deletion of IS*Cg2* family (IS*Cg2b*)	This study
WJ006	ATCC 13032 with in frame deletion of IS*Cg2* family (IS*Cg2b*, IS*Cg2c*)	This study
WJ007	ATCC 13032 with in frame deletion of IS*Cg2* family (IS*Cg2b*, IS*Cg2c*, IS*Cg2e*)	This study
WJ008	ATCC 13032 with in frame deletion of IS*Cg2* family (IS*Cg2b*, IS*Cg2c*, IS*Cg2e*, IS*Cg2f*)	This study
Plasmids		
pCES208	*E. coli—C. glutamicum* shuttle vector, Km^r^	[45]
pCES-NMCS	pCES208 derivative; MCS and *rrn* terminator, Km^r^	[6]
pH36M2	pCES208 derivative; P_H36_, PorB signal sequence, codon-optimized M18 scFv gene (opt)	[5]
pCES-H36-porBss	pCES208 derivative; P_H36_, porinB signal sequence, Km^r^	This study
pCES-H36-porBss-Amy	pCES208 derivative; P_H36_, porinB signal sequence amylase, Km^r^	This study
pCES-H36-porBss-IS-Amy	pCES208 derivative; P_H36_, IS*30* inserted porinB signal sequence, amylase, Km^r^	This study
pCES-H36-GFP	pCES208 derivative; P_H36_, eGFP, Km^r^	[46]
pCnCAB	*phaCABC* gene expression	[41]
pCES-H36-PhaC	pCES208 derivative; P_H36_, *phaC*, Km^r^	This study
pCES-H36-PhaCA	pCES208 derivative; P_H36_, *phaCA*, Km^r^	This study
pCES-H36-PhaCAB	pCES208 derivative; P_H36_, *phaCAB*, Km^r^	This study
pHGmut	pCES208 derivative, Glu89Gln/Δ452-466 gene	[26]
pK19*mobsacB*	Mobilizable vector, Km^r^	[42]
pK19-*∆*IS*Cg1a*	pK19*mobsacB* derivative; flanking region of IS*Cg1a*	This study
pK19-*∆*IS*Cg1b*	pK19*mobsacB* derivative; flanking region of IS*Cg1b*	This study
pK19-*∆*IS*Cg1c*	pK19*mobsacB* derivative; flanking region of IS*Cg1c*	This study
pK19-*∆*IS*Cg1e*	pK19*mobsacB* derivative; flanking region of IS*Cg1e*	This study
pK19-*∆*IS*Cg2b*	pK19*mobsacB* derivative; flanking region of IS*Cg2b*	This study
pK19-*∆*IS*Cg2c*	pK19*mobsacB* derivative; flanking region of IS*Cg2c*	This study
pK19-*∆*IS*Cg2e*	pK19*mobsacB* derivative; flanking region of IS*Cg2e*	This study
pK19-*∆*IS*Cg2f*	pK19*mobsacB* derivative; flanking region of IS*Cg2f*	This study
pJET1.2/blunt	Commercial cloning vector	Thermo^b^

^a^New England Biolabs, Beverly, MA, USA

^b^Thermo fisher scientific, Waltham, MA, USA

### Fluorescence assay and screening by FACS

*C. glutamicum* harboring pCES-H36-GFP was cultivated in BHI medium for 24 h. Optical density of cells were measured and amount of OD_600_ 1 cells were harvested by centrifugation at 6,000 rpm for 10 min. at 4 °C. After washing with 1x phosphate-buffered saline (PBS), cells were resuspended in the same buffer, and the fluorescence intensity was measured with a fluorescent activated cell sorter (FACS; MoFlo XDP, Beckman Coulter, Inc., MI, FL, USA). The cells were excited with a laser at 488 nm and detected with 530/40 band-pass filter for the GFP emission spectrum. Cells showing low fluorescence intensity (mean fluorescence value of <30) were sorted and dropped into fresh BHI media. The cells were grown overnight and transferred into BHI media in a 250 mL flask for the next round of screening.

### Plasmid construction

The enzymes for the recombinant DNA work were purchased from Enzynomics (Daejeon, Korea). Polymerase chain reaction (PCR) was performed with the C1000™ Thermal Cycler (Bio-Rad, Hercules, CA, USA) and the Prime STAR HS polymerase (Takara Bio Inc., Shiga, Japan). All oligonucleotides used for PCR are listed Additional file 5: Table S1. For construction of the secretory production system, the H36 promoter and PorB signal peptide gene were amplified from pH36M2 by PCR with the primers H36-porB-F and H36-porB-R. The PCR product was digested with *Sal*I and *Xba*I and cloned into pCES-NMCS digested with the same restriction enzymes yielding pCES-H36-porBss. The α-amylase gene (GenBank No. AB000829.1) was obtained from the chromosomal DNA of *Streptococcus bovis* by PCR with the primers Amy-F and Amy-R. After digestion of the PCR product with *Sfi*I, the digested product was cloned into pCES-H36-porBss yielding pCES-H36-porBss-Amy. For the production of poly(3-hydroxybutyrate) [P(3HB)], the engineered *Pseudomona* sp. 6–19 PHA synthase (PhaC1*Ps*_6-19_) [40], PhaA, and PhaC genes were amplified from pCnCAB [41] by PCR with three primer sets, ‘PhaC-F and PhaC-R’, ‘PhaA-F and PhaA-R’, and ‘PhaB-F and PhaB-R’, respectively. The PCR product of PhaC was cut with *Bam*HI and *Xba*I, and ligated into pCES-NMCS digested with the same restriction enzymes to yield pCES-H36-PhaC. The PCR product of PhaA was cut with *Xba*I and *Not*I and ligated into pCES-H36-PhaC to yield pCES-H36-PhaCA. The PCR product of PhaB was cut with *Not*I and ligated into pCES-H36-PhaCA to yield pCES-H36-PhaCAB.

To construct the IS element-inserted vector, the IS element was amplified from the chromosomal DNA of *C. glutamicum* by PCR with IS-amy-F and IS-amy-R1, and PCR overlapping was performed with IS-amy-F and IS-amy-R2 using this PCR product. The PCR product was cut with *Bam*HI and *Xba*I and cloned into pCES-H36-porBss-Amy to yield pCES-H36-porBss-IS-Amy. To construct the IS element deletion plasmid pK19-*∆*IS*Cg1a*, the upstream region (called A region) and downstream region (called B region) were amplified with the oligonucleotide pairs, ‘IS*Cg1a* A-F and IS*Cg1a* A-R’ and ‘IS*Cg1a* B-F and IS*Cg1a* B-R’, respectively. The PCR products were used as templates for overlap PCR with IS*Cg1a* A-F/IS*Cg1a* B-R. The resulting PCR products were digested with *Sal*I and *Xba*I and cloned into pK19*mobsac*B to yield pK19-*∆*IS*Cg1a*. This cloning method was used to construct the other pK19-*∆*IS*Cg* IS element deletion plasmid series for the rest of IS*Cg1* and IS*Cg2* family genes using the corresponding primers.

### Protein fractionation and analysis

After cultivation, cells were harvested by centrifugation (6000 rpm, 10 min and 4 °C). The cells pellets were re-suspended in 300 μl of phosphate-buffered saline (PBS, 135 mM NaCl, 2.7 mM KCl, 4.3 mM Na2HPO4, 1.4 mM KH2PO4, pH 7.2) and were disrupted by sonication at 9 min at 50 % pulse and 20 % amplitude (Sonic, Vibra cell, USA). Total protein fraction was collected after sonication. After centrifugation of cell lysates at 10,000 rpm for 10 min at 4 °C, soluble proteins were collected from supernatant. The fractionated protein samples were analyzed by SDS-PAGE and Western blot. In the SDS-PAGE analysis, protein samples were loaded on 12 % polyacrylamide gels and after the gel electrophoresis, the gels were stained with Coomassie brilliant blue [50 % (v/v) methanol, 10 % (v/v) acetic acid, 1 g/L Coomassie brilliant blue R-250] for 1 h and destained using a destaining solution [10 % (v/v) methanol, 10 % (v/v) acetic acid]. For Western blotting, the proteins were transferred on to a polyvinyl difluoride membrane (Roche, Rotkreuz, Switzerland) for 2 h at 70 mA by using Bio-Rad transblot apparatus (Bio-Rad). The membrane was incubated with a blocking solution (TBS-T, Tris-buffered saline, 24.7 mM Tris, 137 mM NaCl, 2.7 mM KCl, and 0.5 % Tween-20) with 5 wt % skim milk for 1 h at room temperature. The membrane was incubated with blocking solution containing a horseradish peroxidease (HRP)-conjugated monoclonal anti-FLAG M2 antibody (Sigma Aldrich, St. Louis, MO, USA) for the immune-detection of FLAG-tagged protein. After the incubation, each membrane was washed four times with TBS-T for 5 min and the ECL kit (GE Healthcare Bio-Science AB, Buckinghamshire, UK) was used for protein detection.

### α-Amylase activity assay

After cell cultivation, 1 mL of cultured cell volume was harvested by centrifugation at 13000 rpm for 10 min. Supernatant of cell culture was obtained and diluted 10 or 100 times with PBS. 100 μL of sample was mixed with 100 μL of 200 μg/mL DQ™ starch which is labeled with BODIPY^®^ FL dye (Molecular Probes™, Eugene, OR, USA). After incubation at room temperature in black tube for 20 min, 100 μL of mixture was loaded on 96 well black plate (Corning, Corning, NY, USA). The fluorescence was measured with 495 nm excitation wavelength and 515 nm filter by using a TECAN Infinite M200 Pro ELISA plate reader (Tecan Group Ltd., Männedorf, Switzerland). The fluorescence value was revised with negative control. One unit (U) of α-amylase was defined as the amount of enzyme required to liberate 1.0 mg of maltose from starch in 1 min at pH 6.9 at 20 °C.

### Construction of the IS element-deleted strains

Deletion of the IS element was done with the double crossover method [42]. To delete IS*Cg1a*, *C. glutamicum* was transformed with pK19-*∆*IS*Cg1a* by electroporation. First, screening was done on a kanamycin BHIS (BHI plus 30 g/L sorbitol) plate to isolate the plasmid-integrated clones. Then, the Km-resistant cells were cultivated in BHI medium, and they were spread on a 10 % (*w*/*v*) sucrose LB plate to pop out the integrated plasmid. With the isolated clones, the deletion of the desired IS element was confirmed by colony-PCR with the two primers IS*Cg1a* A-F and IS*Cg1a* B-R. This gene deletion method was used to engineer the other IS element-deleted strains using the corresponding plasmids and primers. After construction of IS element-deleted strains, existence of IS element was confirmed. To check deletion of IS*Cg*1, PCR was performed with two primer sets, Confirm IS*Cg*1 A F, Confirm IS*Cg*1 A R and Confirm IS*Cg*1 B F, Confirm IS*Cg*1 B R. In case of IS*Cg*2, PCR was performed with two primer sets, Confirm IS*Cg*2 A F, Confirm IS*Cg*2 A R and Confirm IS*Cg*2 B F, Confirm IS*Cg*2 B R.

### Inverse PCR

The chromosomal DNA of *C. glutamicum* ATCC 13032 was prepared with the MasterPure™ DNA purification kit (Illumina, SD, CA, USA). The purified chromosomal DNA was cut with the *Not*I restriction enzyme. Inverse PCR was carried out with the Pfu-X polymerase (Solgent, Daejeon, Korea) and two primers, Confirm-IS-F and Confirm-IS-R. The PCR product was cloned into pJET1.2/blunt using the CloneJET PCR cloning kit (Thermo fisher scientific, Waltham, MA, USA). After transformation into *E. coli* XL1-blue, the plasmid was prepared, and the sequence of the PCR product in plasmid was determined by DNA sequencing.

### Analytical procedures

The content of P(3HB) synthesized in *C. glutamicum* was measured by gas chromatography (GC) [43]. An Agilent 6890N GC system (Agilent Technologies, PA, CA, USA) equipped with an Agilent 7683 automatic injector, flame ionization detector, and a fused silica capillary column (ATTM-Wax, 30 m, ID 0.53 mm, film thickness 1.20 mm, Alltech, Deerfield, IL, USA) was used. The dried cells were treated for methanolysis with 15 % sulfuric acid and benzoic acid as an internal standard [44]. After extraction of the polymers, the content of the synthesized polymer was measured by gas chromatography (Agilent, SCL, CA, USA). Cell concentration, defined as the dry cell weight (DCW) per liter of culture broth, was determined as previously described [43]. The residual cell concentration was defined as the cell concentration minus the P(3HB) concentration. The P(3HB) content (wt%) was defined as the percentile ratio of the P(3HB) concentration to the cell concentration.

To determine the concentration of GABA, the culture supernatant was applied to reversed phase high-pressure liquid chromatography (HPLC, LC-20 AD, CTO-20A, SPD-20A; Shimadzu, Japan) equipped with Zorbax Eclipse amino acid analysis (AAA) column (4.6 × 150 mm 3.5-Micron; Agilent, USA). Mobile phase A (10 mM Na_2_HPO_4_—10 mM Na_2_B_4_O_7_, pH 8.2) and mobile phase B (acetonitrile:MeOH:H_2_O 45:45:10 by volume) were applied for separation of the samples. The elution condition was as follows: equilibration (1.9 min., 100 % A), gradient (16.2 min, 0–57 % B; 3.7 min, 57–100 % B), and cleaning (4 min, 100 % B). The column temperature was maintained at 40 °C. Samples were detected with UV at 338 nm.

### Determination of the transformation efficiency

*C. glutamicum* was prepared for transformation as follows: 200 mL of RG media (BHI 40 g, glucose 10 g, beef extract 10 g, sorbitol 30 g per 1 L) were inoculated with cells from an overnight culture grown in BHI at 30 °C with shaking at 200 rpm. When the optical density of the cells reached an OD_600_ of 1, the cells were harvested by centrifugation at 6000 rpm for 10 min. at 4 °C. The cell pellets were washed twice with 10 % ice-chilled glycerol. The final cell suspension in 10 % glycerol was adjusted to an OD_600_ of 10. To determine the transformation efficiency, the following procedure was used [8]. pCES-H36-GFP (500 ng prepared from *C. glutamicum*) was mixed with 150 μL of cell suspension on ice. Electroporation was carried out in a 1 mm cuvette at 1.8 kV, 25 μF, and 200 Ω using a Gene Pulser system (BioRad, Hercules, CA, USA). After the electroporation, 1 mL of RG medium was immediately added to the cells, and the cells were cultivated at 30 °C and 200 rpm for 2 h, and then the cells were plated on RG agar plates containing 25 μg/mL kanamycin. The colony forming units (CFUs) were counted after one day of incubation at 30 °C, and the number of colonies was calculated as the CFU per μg DNA.

## Additional files

10.1186/s12934-015-0401-7 SDS-PAGE analysis of the sorted cells by FACS.
10.1186/s12934-015-0401-7 Confirmation of IS element deletion by agarose gel electrophoresis of PCR samples
10.1186/s12934-015-0401-7 Growth profile of cells harboring pCES-H36-GFP.
10.1186/s12934-015-0401-7 Growth profile of cells harboring pCES-H36-PhaCAB or pHGmut.
10.1186/s12934-015-0401-7 List of primers used in the PCR experiments.

## Abbreviations

IS element: insertion element
FACS: fluorescence cell sorter
GABA: γ-aminobutyrate
P(3HB): poly(3-hydroxybutyrate)
