# Supplementary material for: Enhanced production of recombinant proteins with Corynebacterium glutamicum by deletion of insertion sequences (IS elements)
Source: Microb Cell Fact. 2015 Dec 29;14:207. doi: 10.1186/s12934-015-0401-7 (PMC4696348; doi:10.1186/s12934-015-0401-7)
Supplement: Supplementary file 1 — 10.1186/s12934-015-0401-7 SDS-PAGE analysis of the sorted cells by FACS. [file 12934_2015_401_MOESM1_ESM.pdf]

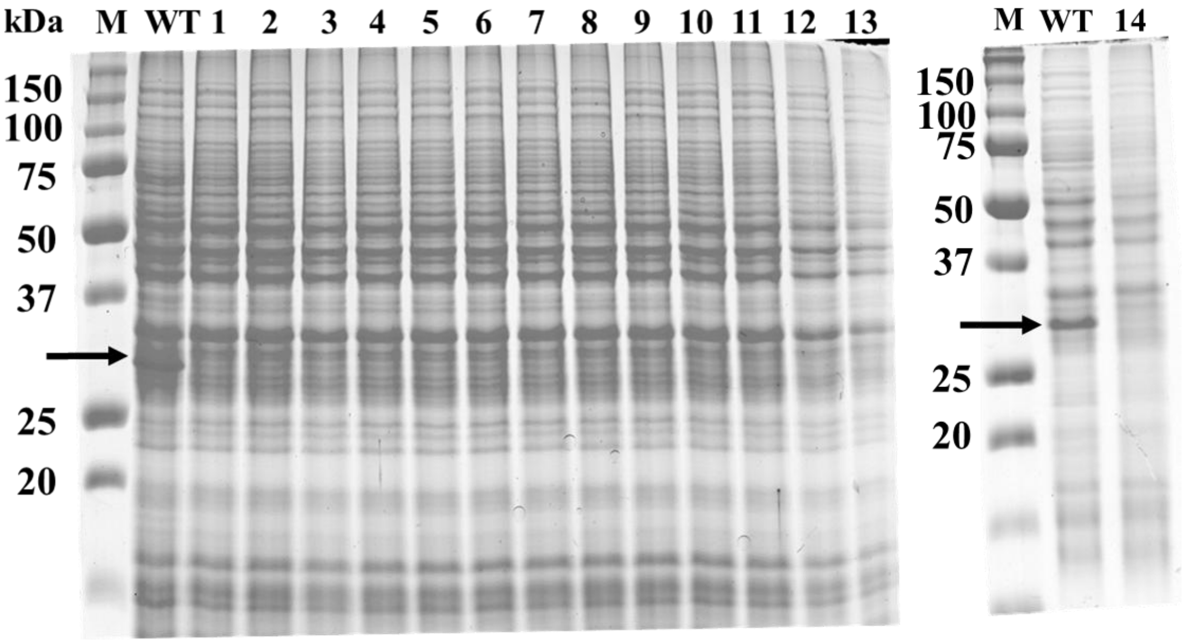

Figure S1. SDS-PAGE analysis of the sorted cells by FACS. After cell sorting, all 14 individual clones were cultured and soluble lysates were prepared by centrifugation following sonication. WT means wild type *C. glutamicum* harboring pCES-H36-GFP. Lanes 1 to 14: clones isolated by FACS screening. Lane M: molecular weight markers (kDa). Arrows indicate the GFP.
