# Supplementary material for: Enhanced production of recombinant proteins with Corynebacterium glutamicum by deletion of insertion sequences (IS elements)
Source: Microb Cell Fact. 2015 Dec 29;14:207. doi: 10.1186/s12934-015-0401-7 (PMC4696348; doi:10.1186/s12934-015-0401-7)
Supplement: Supplementary file 2 — 10.1186/s12934-015-0401-7 Confirmation of IS element deletion by agarose gel electrophoresis of PCR samples [file 12934_2015_401_MOESM2_ESM.pdf]

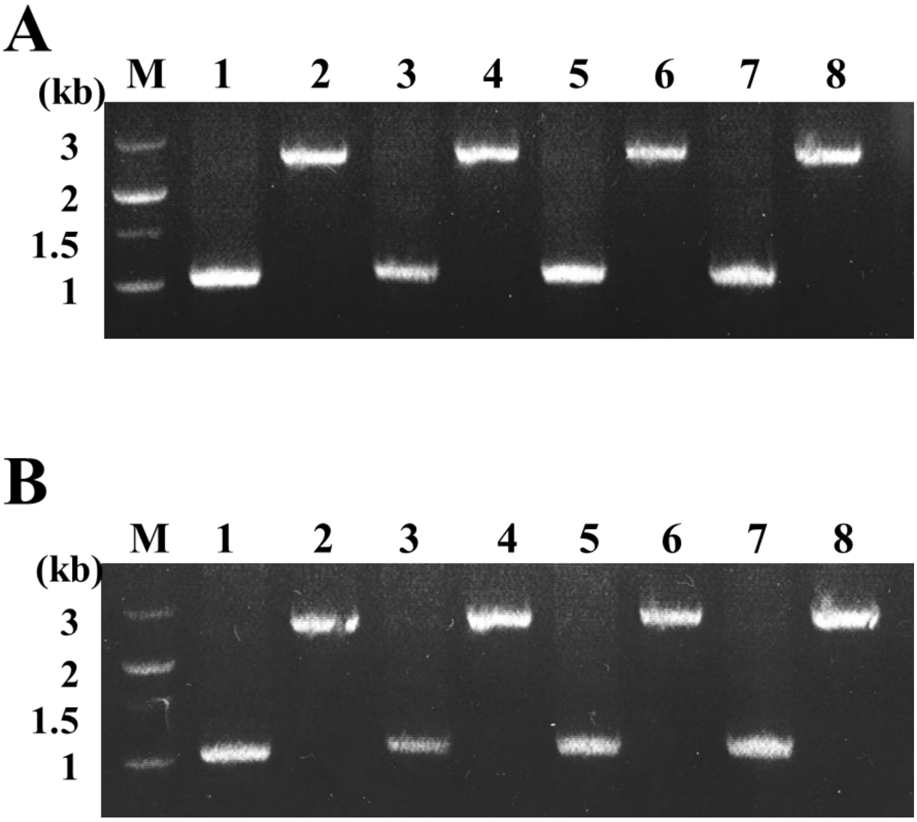

Figure S2. Confirmation of IS element deletion by agarose gel electrophoresis of PCR samples. (A) Confirmation of IS*Cg*1 deletion. Odd number and even number lanes mean PCR product obtained from WJ004 and wild type *C. glutamicum*, respectively. Lanes 1 and 2, IS*Cg1a*; lanes 3 and 4, IS*Cg1b*; lanes 5 and 6, IS*Cg1d*; lanes 7 and 8, IS*Cg1e*. Lane M mean the DNA molecular size markers (kb). In WJ004 strain, IS*Cg1* (~2400 bp) was deleted in each position and the reduced size of PCR product could be obtained. (B) Confirmation of IS*Cg2* deletion. Odd number and even number lanes mean PCR product obtained from WJ008 and wild type *C. glutamicum*, respectively. Lanes 1 and 2, IS*Cg2b*; lanes 3 and 4, IS*Cg2c*; lanes 5 and 6, IS*Cg2d*; lanes 7 and 8, IS*Cg2f*. Lane M mean the DNA molecular size markers (kb). In WJ008 strain, IS*Cg2* (~2600 bp) was deleted in each position and the reduced size of PCR product could be obtained.
