# Supplementary material for: Enhanced production of recombinant proteins with Corynebacterium glutamicum by deletion of insertion sequences (IS elements)
Source: Microb Cell Fact. 2015 Dec 29;14:207. doi: 10.1186/s12934-015-0401-7 (PMC4696348; doi:10.1186/s12934-015-0401-7)
Supplement: Supplementary file 4 — 10.1186/s12934-015-0401-7 Growth profile of cells harboring pCES-H36-PhaCAB or pHGmut. [file 12934_2015_401_MOESM4_ESM.pdf]

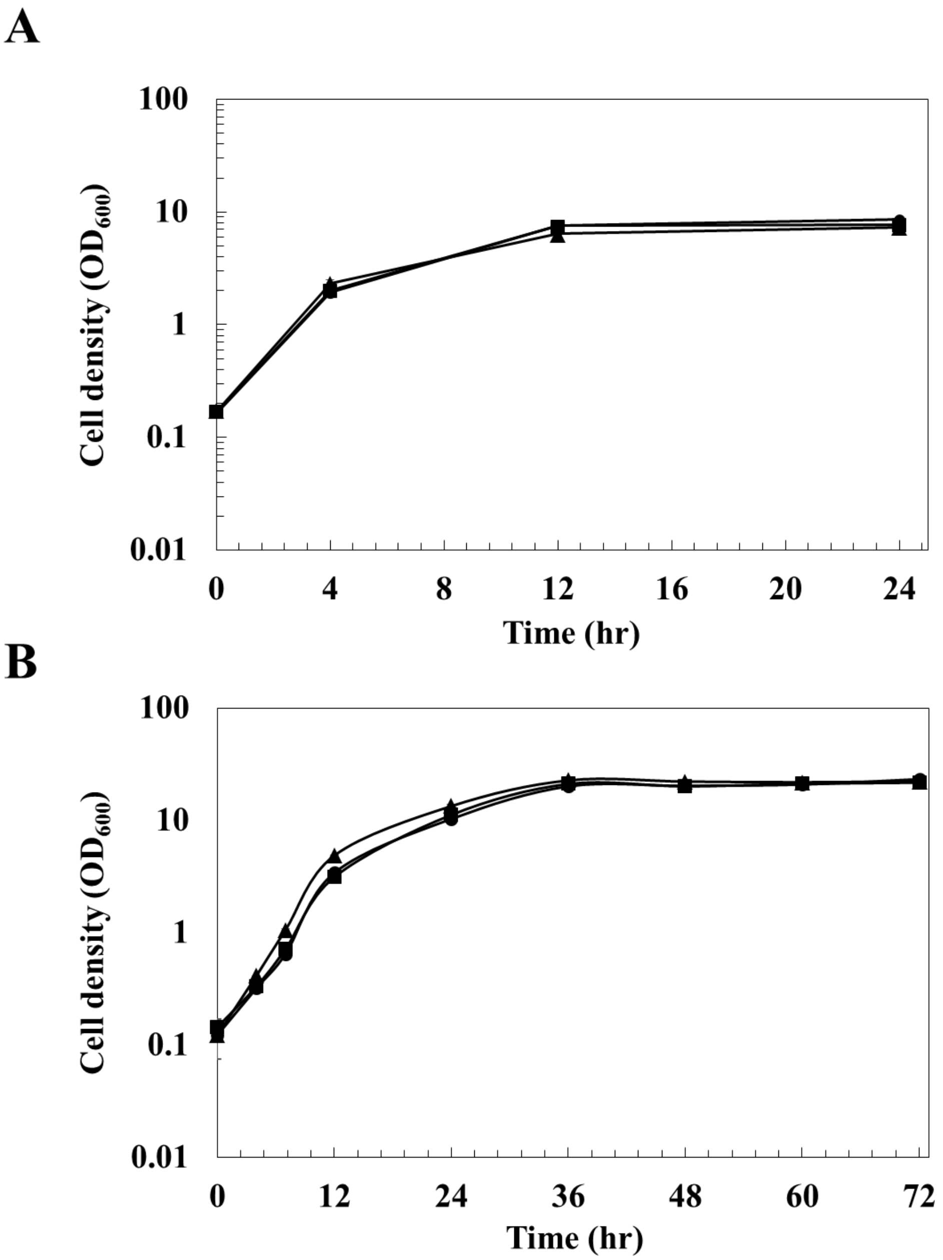

Figure S4. Growth profile of cells harboring pCES-H36-PhaCAB or pHGmut. (A) *C. glutamicum* WJ004 harboring pCES-H36-PhaCAB and *C. glutamicum* WJ008 harboring pCES-H36-PhaCAB are represented by squares (■) and circles (●), respectively. Triangles (▲) represent wild type *C. glutamicum* harboring pCES-H36-PhaCAB, which was used as a positive-control. (B) *C. glutamicum* WJ004 harboring pHGmut and *C. glutamicum* WJ008 harboring pHGmut are represented by squares (■) and circles (●), respectively. Triangles (▲) represent wild type *C. glutamicum* harboring pHGmut, which was used as a positive-control
