# Supplementary material for: Enhanced production of recombinant proteins with Corynebacterium glutamicum by deletion of insertion sequences (IS elements)
Source: Microb Cell Fact. 2015 Dec 29;14:207. doi: 10.1186/s12934-015-0401-7 (PMC4696348; doi:10.1186/s12934-015-0401-7)
Supplement: Supplementary file 5 — 10.1186/s12934-015-0401-7 List of primers used in the PCR experiments. [file 12934_2015_401_MOESM5_ESM.pdf]

Table S1. List of primers used in the PCR experiments.

Primer name	Primer sequence (5' to 3')[a]
H36 porB F	ATA**GTCGAC**GGTACCTCTATCTGGTGCC
H36 porB R	GTGTCAT**TCTAGA**TGCGGAAGCAGGTGCT
Amy F	ATTAAT**GGCCCAGCCGGCC**AAGATGAACAAGTGTCAATGAAAGATG
Amy R	ATTAAT**GGCCCCCGAGGCC**CTATTAATGATGGTGATGGTGATGTTTTAGCCCATCTTTATTATAGTTTCCAGAT
IS amy F	TGACAC**GGATCC**ATGAAGCTTTCACACCGCATCGATGTCAGGTCTTGCTGCG
IS amy R 1	GCTGCCACTGTGATGCCTGCGGTTGCTGCCATTGCTGTCAATCGGTGGATGCACC
IS amy R 2	GCTGTCA**TCTAGA**TGCGGAAGCAGGTGCTGCGAATGCTGCCACTGTGATGCC
IS*Cg1a* A-F	TACGAC**GTCGAC**CACTTCCAACTGGCACGTT
IS*Cg1a* A-R	GGTTTACGGGCTCTTCCTGTTGGGTAGAGCCTTTTGTTGGTGT
IS*Cg1a* B-F	ACACCAACAAAAGGCTCTACCCAACAGGAAGAGCCCGTAAACC
IS*Cg1a* B-R	CGTCGA**TCTAGA**TGGTCAAAGCTTCCCCTGG
IS*Cg1b* A-F	ATCCAG**GTCGAC**CCACTGTCTTCGAAGCACAAC
IS*Cg1b* A-R	GCTCTTAAGTGGCTCTTCCTGTTGGGTAGAGCCTTTTGTTGGTGT
IS*Cg1b* B-F	ACACCAACAAAAGGCTCTACCCAACAGGAAGAGCCACTTAAGAGC
IS*Cg1b* B-R	GTGCTA**TCTAGA**CGGGCAAGCACACGTC
IS*Cg1d* A-F	TAGCAC**GTCGAC**CCCATCTTTGTGGTGGCTG
IS*Cg1d* A-R	ATACGTTTACTGGCTCTTCCTGTTGGGTAGAGCCTTTTGTTGGTG
IS*Cg1d* B-F	CACCAACAAAAGGCTCTACCCAACAGGAAGAGCCAGTAAACGTAT
IS*Cg1d* B-R	GTGCTA**TCTAGA**AATCATCACCTCCCGTGAAG
IS*Cg1e* A-F	TGACCAC**CTGCAG**AAGTCAACGACCGCAAGC
IS*Cg1e* A-R	CTGCCCCACAAATAAAAAACACCGCGAAGCAGAAACTGC
IS*Cg1e* B-F	GCAGTTTCTGCTTCGCGGTGTTTTTTATTTGTGGGGCAG

IS*Cg1e* B-R	GTGTCA**TCTAGA**GTTCATCATTGCGGTCGACA
IS*Cg2b* A-F	TAGCAC**GTCGAC**TCATGGTTCAGGGCACTG
IS*Cg2b* A-R	TCGTACAATCTCCTAGGCGAATACCTTGATTGATCATGTCGAGG
IS*Cg2b* B-F	CCTCGACATGATCAATCAAGGTATTCGCCTAGGAGATTGTACGA
IS*Cg2b* B-R	TCGTGA**TCTAGA**CTGCTCATGATTTCCCGCA
IS*Cg2c* A-F	TAGCAC**GTCGAC**GCGCCCTGGCAATGC
IS*Cg2c* A-R	CGGAACTGACGGCGAATACCTTGATTGATCATGTCGAGGAAA
IS*Cg2c* B-F	TTTCCTCGACATGATCAATCAAGGTATTCGCCGTCAGTTCCG
IS*Cg2c* B-R	GTCTGA**TCTAGA**GTCTCCTAGGCGTTCCTACT
IS*Cg2d* A-F	TGACAC**GTCGAC**TTTCGTGATCCTGGGTTGG
IS*Cg2d* A-R	GCATAATAGGGACGGCGAATACCTTGATTGATCATGTCGAGGAAA
IS*Cg2d* B-F	TTTCCTCGACATGATCAATCAAGGTATTCGCCGTCCCTATTATGC
IS*Cg2d* B-R	GTGCTA**TCTAGA**ATCACTCACCATCATCGGC
ISCg2*f* A-F	TAGCAC**GTCGAC**ACTGCCCCCTCTGGAAATG
ISCg2*f* A-R	CATCCAACCTAGGGCGAATACCTTGATTGATCATGTCGAGG
ISCg2*f* B-F	CCTCGACATGATCAATCAAGGTATTCGCCCTAGGTTGGATG
ISCg2*f* B-R	GTGTCA**TCTAGA**CGATGGAATAATCAGACTCTGGAAC
Confirm IS*Cg*1 A F	ATGAAGTCTACCGGCAACAT
Confirm IS*Cg*1 A R	TCCACCCCAATGACATACAC
Confirm IS*Cg*1 B F	GCCGGCAACGCCT
Confirm IS*Cg*1 B R	TTAGAGTGCATTGATCTTATGGACC
Confirm IS*Cg*2 A F	ATGTCAGGTCTTGCTGCG
Confirm IS*Cg*2 A R	TTGATTTCATCAGCAAATAACGGCA
Confirm IS*Cg*2 B F	CAGAAGTTGCTGATCGTGCT
Confirm IS*Cg*2 B R	TCAATCGGTGGATGCACC
PhaC F	A**GGATCC**ATGGCGACCGGCAAAGG
PhaC R	C**TCTAGA**TCACCGTTCGTGCACG
PhaA F	ATGCTA**TCTAGA***AAAGGAGGAAAATC*ATGACTGACGTTGTCATCGTATC
PhaA R	GATGCA**GCGGCCGC**TTATTTGCGCTCGACTGCCA
PhaB F	CGATAC**GCGGCCGC***AAAGGAGGAAAATC*ATGACTCAGCGCATTGCG
PhaB R	AGAT**GCGGCCGC**TCAGCCCATATGCAGGC

[a] Restriction enzyme sites and ribosome binding sites are shown in bold and italic.
